# Fibronectin in the Tumor Microenvironment Activates a TLR4-dependent Inflammatory Response in Lung Cancer Cells

**DOI:** 10.7150/jca.39771

**Published:** 2020-03-04

**Authors:** Christina Cho, Carol Horzempa, Christine M. Longo, Donna M. Peters, David M. Jones, Paula J. McKeown-Longo

**Affiliations:** 1 Department of Regenerative & Cancer Cell Biology, Albany Medical College, 47 New Scotland Avenue, Albany, NY 12208-3479; 2 Department of Pathology & Laboratory Medicine, University of Wisconsin, 1300 University Avenue, Madison, Wisconsin 53706; 3 Department of Pathology & Laboratory Medicine, Albany Medical College, 47 New Scotland Avenue, Albany, NY 12208-3479

**Keywords:** tumor microenvironment, IL-8, fibronectin, toll-like receptor, extracellular matrix

## Abstract

The microenvironment of solid tumors plays an essential role in tumor progression. In lung cancer, the stromal cells produce a fibronectin rich extracellular matrix which is known to contribute to both tumor metastasis and drug resistance. Due to its conformational lability, fibronectin is considerably remodeled by the contractile forces of the fibrotic microenvironment within the tumor stroma. As a result, the secondary structure of fibronectin's Type III domains is disrupted and the molecule becomes highly stretched. The contribution/impact of these strained forms of fibronectin on tumor growth and metastasis is not known. In the current study we show that the partially unfolded first Type III domain of fibronectin, III-1c, activates a toll-receptor/NF-κB pathway leading to an increase in the expression of IL-8. Using a 3-D model of tumor-associated extracellular matrix, we demonstrate that lung cancer cells seeded onto this matrix activate a TLR4/NF-κB signaling pathway leading to a robust increase in the release of IL-8. Cytokine release by these cells is completely dependent on the presence of fibronectin in the extracellular matrix. These findings suggest that paracrine signaling between the tumor and the stromal myofibroblasts causes a remodeling of the matrix fibronectin into a strained conformation which supports the activation of a TLR4/NF-κB signaling pathway resulting in the upregulation of fibro-inflammatory cytokines.

## Introduction

Fibronectin matrix is upregulated in the stroma of human non-small cell lung cancer (NSCLC) where it has been shown to promote cancer cell growth, migration, invasion, survival, and resistance to chemotherapy. Consequently, the molecular pathways controlling the tumor cell response to the stromal fibronectin matrix are now regarded as potential drug targets for the management of chemo-resistant tumors 1; however, the molecular pathways regulated by fibronectin present in the stroma of solid tumors remain poorly understood. Fibronectin is synthesized by cancer associated fibroblasts and polymerized into extracellular matrix (ECM) fibrils which serve as scaffolds for the binding of growth factors, other ECM molecules and cell surface receptors 2. Structurally, fibronectin is organized into individually-folded domains, some of which (Type III) unfold in response to increased mechanical forces. The unfolding of the Type III domains allows fibronectin fibers to stretch up to several times their length, thus altering the availability of various functional domains 3,4. Mechanically regulated activities of matrix fibronectin include polymerization of soluble fibronectin and the binding of integrins, collagen, bacterial adhesins, and growth factors 5.

Recent studies have shown fibronectin in the tumor stroma to be highly stretched due to the unfolding of Type III domains 6,7. How these tumor-driven changes in fibronectin structure impact cancer progression is not well understood. To address this question, we have made use of a fibronectin peptide mimetic, FnIII-1c, which corresponds to a stable intermediate structure predicted to form during force-induced unfolding of the first Type III domain of fibronectin 8. In previous studies, we have identified FnIII-1c as a Damage Associated Molecular Pattern (DAMP) molecule, which activates Toll-like receptors (TLRs) to initiate NF-κB activation and inflammatory cytokine release in fibroblast cells 9,10.

Interleukin-8 (IL-8) is a pro-inflammatory cytokine which is upregulated in many types of cancer including lung cancer. IL-8 influences lung tumorigenesis by promoting mesenchymal and stem cell phenotypes, angiogenesis, chemoresistance and suppression of anti-tumor immunity 11. We have also shown that lung tumors are enriched in fibronectin and IL-8 12, consistent with fibronectin in the tumor matrix promoting the TLR dependent release of cytokines by lung cancer cells. In the present study, we used an established 3D model of decellularized tumor-associated ECM containing stretched and unfolded fibronectin 6,7 and evaluated the impact of this 3D matrix on IL-8 induction in NSCLC cells. The data indicate that NSCLC cells treated with FnIII-1c or seeded onto tumor-associated, but not control ECM, upregulate IL-8 production. We further show that the release of IL-8 by NSCLC cells seeded onto tumor-associated ECM is dependent on fibronectin, NF-κB and TLR4. Additionally, we evaluated a panel of human NSCLC tumors and found that 32/33 tumors stained positive for active NF-κB. Taken together, the data are consistent with a model in which tumor-dependent remodeling of the fibronectin in the ECM promotes inflammation through the activation of TLR4/NF-κB signaling in NSCLC.

## Materials and Methods

### Antibodies and reagents

Unless indicated otherwise, all reagents were purchased from Sigma (St. Louis, MO). Fetal bovine serum (FBS) was from Hyclone (Logan, UT). Monoclonal mouse antibodies against phospho-p38 MAP- kinase, NFκB and GAPDH were from Cell Signaling Technology (Danvers, MA). Antibodies to phospho- JNK (Thr183/185), and lamin A/C were from Santa Cruz Biotechnology, Inc. (Santa Cruz, CA). Antibody to NF-κB phospho (S536) p65 used for tissue array staining was from Abcam (Cambridge, MA). Alexafluor488-conjugated secondary anti-mouse IgG (H+L) antibody was from Molecular Probes (Eugene, OR). Horseradish peroxidase (HRP) conjugated secondary antibodies to mouse IgG (H+L) and rabbit IgG (H+L) were from BioRad (Berkeley, CA). Antibodies against the 40kDa (gelatin-binding) region of fibronectin were described previously 13. The functional upstream domain (FUD) of streptococcus pyogenes adhesin F1 protein was described previously 14. The TLR4 (TAK-242) and NF-κB (BAY11-7082) inhibitors were from EMD Millipore (Temecula, CA). Recombinant His-tagged fibronectin Type III domains were described previously 15.

### Cell culture

Human NSCLC cell lines NCI-H460, Calu-1 and A549 were from the American Type Culture Collection (ATCC, Manassas, VA). Cells were cultured in either RPMI-1640 (H460), McCoy's 5A (Calu1) or F12 (A549) supplemented with 10% FBS, streptomycin-penicillin and glutamax. Cell monolayers were washed in ice-cold PBS, lysed in whole cell lysis buffer (100mM Tris-HCl, pH 6.8, 2% SDS, 10% glycerol, 100 mM DTT), and lysates analyzed by Western blot 16. Immunoreactive bands were detected with Clarity^TM^ Western ECL Blotting Substrates (BioRad, Berkeley, CA) and quantified using the BioRad ChemiDoc™ MP imaging system with Image Lab^TM^ Software (6.0).

3T3-L1 mouse pre-adipocytes (ATCC) were cultured in MEM (α-modification [α-MEM] Sigma-Aldrich) containing 10% FBS glutamax and streptomycin-penicillin and utilized at passage 8 or lower. MDA-MB-231 human breast cancer cells (ATCC) were cultured in DMEM (Dulbecco's modified eagle medium; Sigma-Aldrich) containing 10% FBS, streptomycin-penicillin and glutamax. For the collection of tumor-associated medium, DMEM was replaced with MEM (α-modification [α-MEM], Sigma-Aldrich) containing 1% fetal bovine serum, glutamax and streptomycin-penicillin. All cells were maintained at 37ºC in a humidified atmosphere containing 5% CO_2_**.**

### Preparation and collection of tumor-associated medium

Tumor-conditioned and control media were prepared as previously described 6. MDA-MB-231 cells (ATCC) (80% confluent) were incubated in αMEM with streptomycin-penicillin and glutamax supplemented with 1% FBS for 24 hours. To prepare control medium, 15 mL of 1% FBS-αMEM with streptomycin-penicillin and glutamax were incubated in cell-free tissue culture plates for 24 hours. Tumor-conditioned and control media were clarified by centrifugation and concentrated 10-fold using Amicon Ultra-free 15 centrifugal filter units (3000 MWCO, Millipore). The concentrated media were diluted two-fold by volume with 1% FBS-αMEM containing streptomycin-penicillin and glutamax.

### Generation of decellularized 3D tumor-associated and control matrices

A 3D model of tumor-associated decellularized matrix was prepared as previously described 7. In this model, 3T3-L1 pre-adipocytes are cultured in medium conditioned by MDA-MB-231 breast cancer cells to mimic paracrine signaling between a tumor and its stromal microenvironment. 3T3-L1 cells were plated onto gelatin-coated 3.5 cm^2^ tissue culture plates at 3000 cells/cm^2^ and incubated in 10%FBS-αMEM with streptomycin-penicillin and glutamax at 37ºC until ~85% confluent. The culture medium (10%FBS-αMEM) was replaced with either control medium or tumor-conditioned medium which were both supplemented with 50 µg/ml ascorbic acid (Sigma). Control and tumor-conditioned media were replaced every other day. To prevent fibronectin assembly into the ECM, 3T3-L1 cells were seeded in the presence of 2 μm FUD. Cells were grown for 6 days and medium containing FUD was changed every other day. Recombinant FUD was prepared as previously described 14. On day 8, 3D decellularized matrix was prepared by solubilizing 3T3-L1 cells in 1 mL of extraction buffer (20 mM NH_4_OH and 0.5% Triton-X in 1X PBS) for 5 minutes. 2 mL of pre-warmed (37ºC) PBS was then added to each plate and the plates were incubated at 4ºC overnight. Decellularized matrices were prepared by removal of extraction buffer and thorough washing of the matrices with PBS. NCI-H460 and A549 cells (~10^5^) were seeded onto decellularized control and tumor-associated 3D matrices and incubated in serum-free media supplemented with 0.1% BSA. At the designated times, conditioned media were collected and IL-8 levels were determined by ELISA.

### Immunofluorescence

3T3-L1 cells were seeded onto gelatin-coated glass coverslips (18 mm diameter) at a density of 3000 cells/cm^2^ then cultured as described above. On day 8, coverslips were rinsed once with PBS and fixed in 4% paraformaldehyde in PBS for 20 minutes. Fixed cells were permeabilized in 0.5% TritonX-100 in PBS for 10 minutes then treated with 0.1 M glycine in PBS for 10 minutes at room temperature. Next, cells were blocked in 1% BSA, 0.3% TritonX-100 in PBS, immunostained with an antibody against human fibronectin followed by Alexa Fluor488-conjugated secondary anti-rabbit antibody (5 µg/mL). Actin was stained with phalloidin (1:10,000). Stained cells were counterstained with Hoechst 33342 (1 µg/mL), mounted with Prolong Antifade Molecular Probes and examined using an Olympus BMX-60 microscope equipped with a cooled CCD sensi-camera (Cooke, Auburn Hills, MI). Images were acquired using Slidebook software (Intelligent Imaging Innovation, Denver, CO).

### Tissue Section Staining

NSCLC tissue microarray panels (LC121c US Biomax, Inc., Rockville, MD) were immuno-stained using the peroxidase-based ABC system (Vector Laboratories, Burlingame, CA). The activated subunit of NF-κB, phosphorylated p65/rel A subunit was detected using a rabbit polyclonal antibody (p65/relA phosphoS536), as primary antibody. Negative control sections received no primary antibody. Color was developed by reaction with 3,3'-diaminobenzidine. Tissue sections were counterstained with hematoxylin.

## Results and Discussion

### Fibronectin's unfolded III-1 domain activates TLR4 signaling and IL-8 release in NSCLC

**Figure 1:**
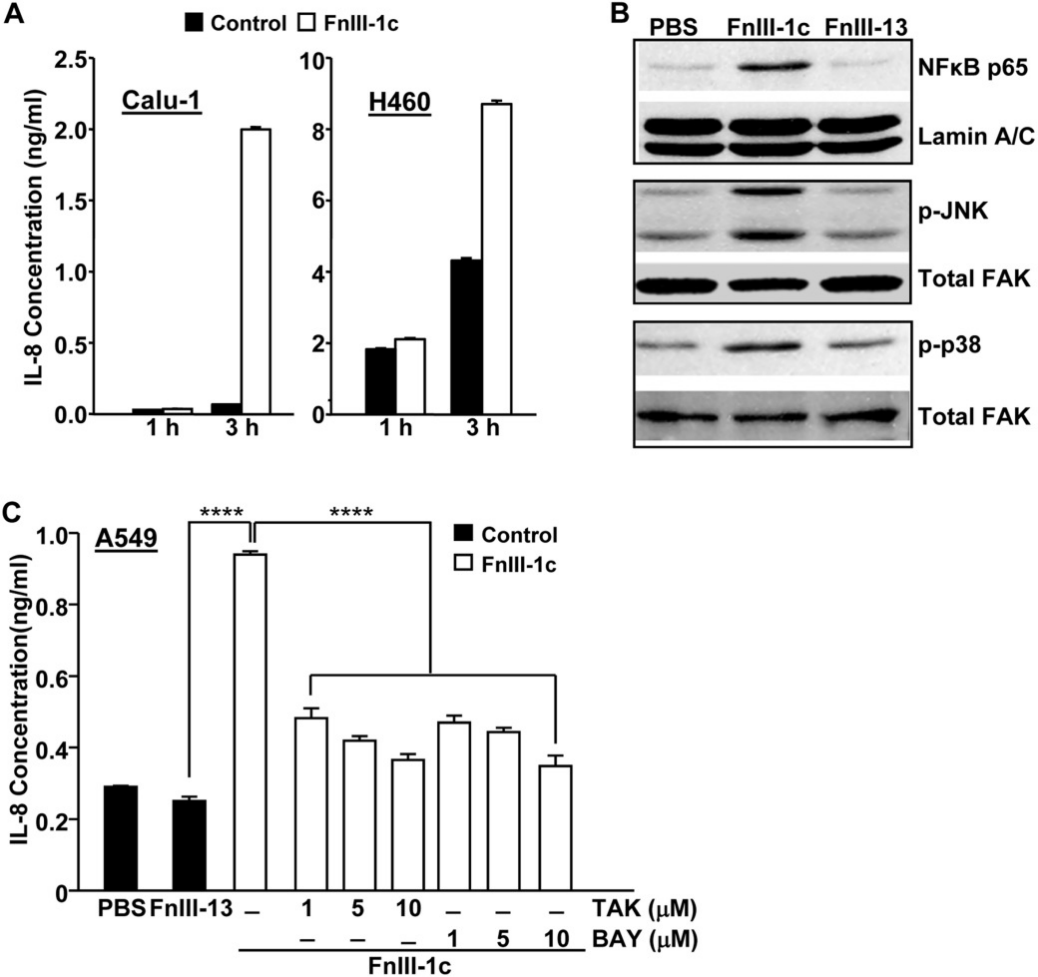
** FnIII-1c induces TRL4-dependent IL-8 expression in NSCLC cells.** (**A**) Calu-1 and H-460 cell monolayers in serum-free medium were incubated with 10 μM FnIII-1c or PBS (control) for the designated times. Conditioned medium was assayed for IL-8 by ELISA. (**B**) Calu-1 human lung cancer cells were treated with 20 µM FnIII-1c, 20 µM FnIII-13 (control) or PBS in 0.1% BSA/DMEM (Vehicle control) for 1 hour. The nuclear fraction was isolated and analyzed by Western blot for the presence of the NF-κB subunit p65/relA. The membranes were then stripped and reprobed with an antibody against nuclear Lamin A/C as loading control. The cytosolic fraction was electrophoresed and immunoblotted using antibodies against phosphorylated JNK (p-JNK) or phosphorylated p38 (p-p38). The membranes were then stripped and reprobed with antibodies against FAK as loading control. (**C**) NSCLC cells (A549) were incubated with 20 µM FnIII-1c, PBS or 20 μM FnIII-13 (control) for 6 hours in the presence or absence of inhibitors of TLR4 (TAK) or NF-κB (BAY). Error bars represent the mean ± SE of triplicate samples from one representative experiment. ****(P<0.0001).

We have previously shown that a fibronectin- derived peptide representing the unfolded III-1 domain of fibronectin, FnIII-1c, induced an inflammatory response in human-dermal and adult lung fibroblasts 10,12. FnIII-1c mediated cytokine release was induced through TLR/NF-κB/p38- dependent signaling pathways. As other studies have also implicated an NF-κB/p38/JNK dependent pathway in the regulation of cytokine expression in pulmonary inflammation 17, we assessed whether FnIII-1c could activate these same signaling pathways in NSCLC cells. As shown in Figure 1A, the addition of FnIII-1c to Calu-1 cells induced a 10-fold increase in IL-8 protein levels within 3 hours. Similarly, an FnIII-1c-dependent increase in IL-8 production was also seen in H460 cells. Western blot analysis of nuclear and cell lysates from Calu-1 cells, which had been treated with FnIII-1c for 1 hour, showed an increase in nuclear localization of NF-κB and in the phosphorylation of both JNK and p38. These findings are consistent with FnIII-1c causing an activation of NF-κB and MAP kinase signaling (Fig. 1B). A control Fn-III domain, III-13, had no effect on either NF-κB nuclear localization or MAP kinase activation. Incubation of a third NSCLC cell type, A549, with FnIII-1c resulted in a 3-fold increase in the synthesis of IL-8, which was prevented by inhibitors of TLR4 (TAK-242) and NF-κB (BAY 11-7082) (Fig. 1C). These data indicate that similar to our previous findings in fibroblasts 10,15. FnIII-1c also activated a TLR4/NF-κB signaling pathway in NSCLC cells that resulted in a rapid (<3 hours) release of IL-8. Further studies are needed to determine whether FnIII-1c binds directly to TLR4 or activates TLR4 signaling through ancillary molecules functioning as co-receptors 9. IL-8 plays several important roles in the progression of lung cancer including promoting an inflamed microenvironment, modulating the response to chemotherapy and serving as a diagnostic or prognostic indicator of both disease progression and drug resistance 18.

### IL-8 secretion by NSCLC is upregulated by fibronectin in the tumor-associated ECM

**Figure 2:**
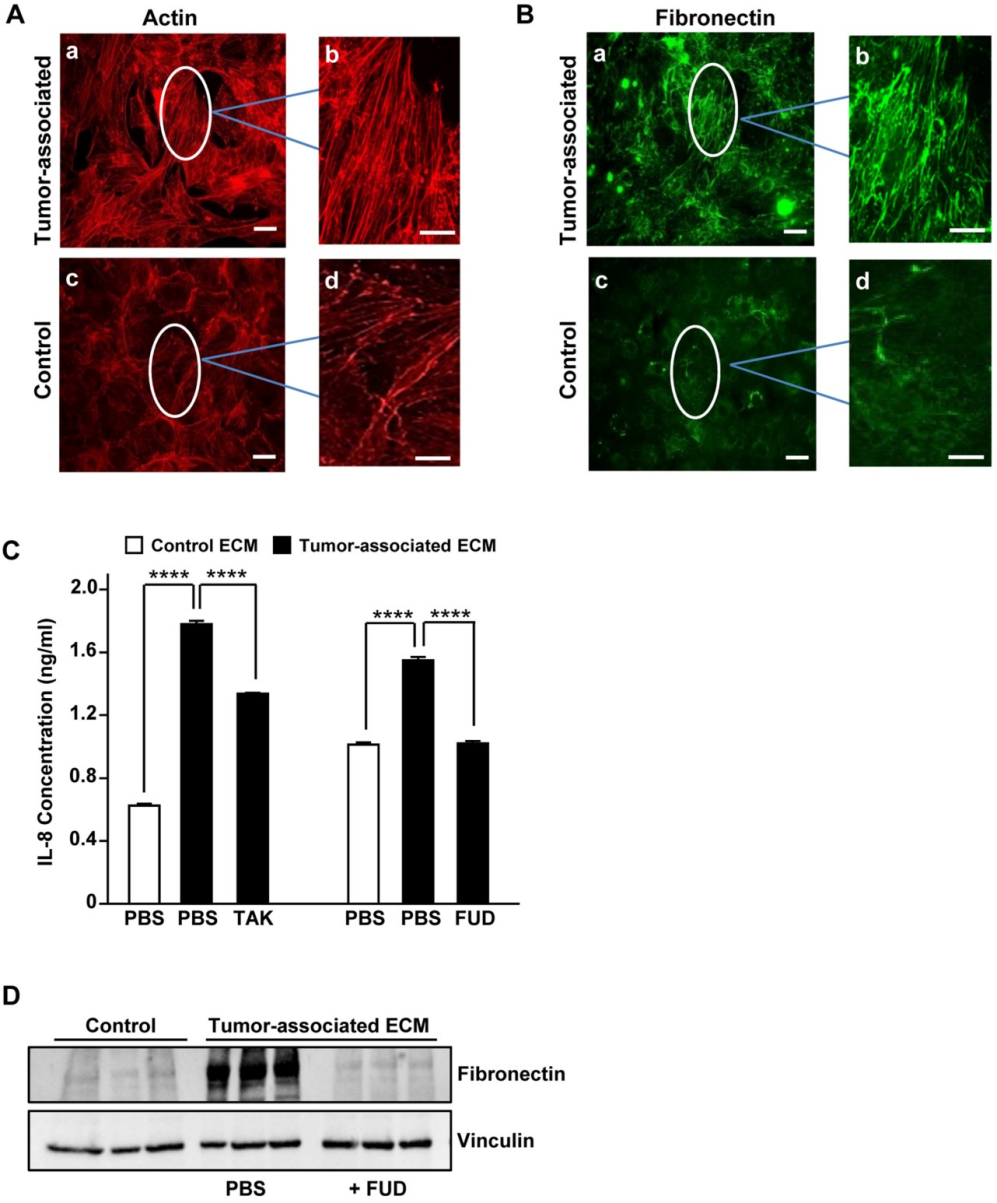
** Tumor-associated fibronectin matrix induces IL-8 expression in human NSCLC cells. (A)** 3T3-L1 pre-adipocytes were cultured in either control medium or tumor-conditioned medium for one week. Cell monolayers were fixed, permeabilized and stained for actin with phalloidin or (**B**) immunostained for fibronectin. The designated areas in panels a and c (scale bar = 50 µm.) were enlarged (panels b and d) to visualize fibronectin matrix and actin fibers. Scale bar = 10 µm. (**C**) A549 cells were seeded in serum-free medium onto either decellularized control (x) or tumor-associated ECM (f) in the presence of the inhibitor (10 μM TAK) of TLR4 signaling or the inhibitor (2 μM FUD) of fibronectin matrix assembly. PBS served as control. After an overnight incubation, conditioned media were collected and assayed for IL-8 by ELISA. (**D**) Fibronectin levels in control matrix, tumor-associated matrix or tumor-associated matrix prepared in the presence of FUD (2 μM) was analyzed by Western blot. Vinculin served as loading control. ****P<0.0001.

To evaluate whether tumor-associated ECM could impact IL-8 expression in NSCLC, we made use of an established 3-D model of decellularized tumor-associated ECM. In this model, paracrine signaling between pre-adipocytes (3T3-L1 cells) and breast cancer cells (MDA-MB-231) results in the differentiation of adipocytes into myofibroblasts which deposit a robust fibronectin matrix characterized by stretched and unfolded fibrils 6,19,7. As previously described 6,7, phalloidin staining of 3T3-L1 cells cultured in breast tumor-conditioned medium revealed well organized actin-stress fibers characteristic of myofibroblasts (Fig. 2A, panels a,b). Earlier studies have shown that the increase in stress fibers is associated with increased cellular contractile force and matrix rigidity 7,20. In contrast, actin stress fibers were less prominent in 3T3-L1 cells cultured in control medium (Fig. 2A, panels c,d). Consistent with the myofibroblast phenotype, 3T3-L1 cells grown in tumor-conditioned medium also assembled a prominent fibronectin matrix containing linearly aligned fibers (Fig. 2B, panels a, b). In contrast, 3T3-L1 cells grown in control medium exhibited diffusely stained and less well-organized fibronectin (Fig. 2B, panels c, d).

To determine whether tumor-associated ECM could induce an increase in IL-8 synthesis in NSCLC cells, A549 cells were seeded onto decellularized 3D control or tumor-associated ECM. Following an overnight incubation, conditioned media were analyzed for IL-8 by ELISA. As shown in Fig. 2C, A549 cells seeded onto 3D tumor-associated ECM exhibited an increase in IL-8 expression which was partially blocked by TAK-242, an inhibitor of TLR4 signaling. To assess the role of matrix fibronectin in mediating this increase in IL-8 expression, tumor- associated ECM was prepared under conditions where fibronectin polymerization was inhibited. Assembly of fibronectin into the ECM can be prevented by fibronectin binding peptides such as the functional upstream domain (FUD) of the *Streptococcus pyogenes* adhesion F1 protein 21. As shown in Fig. 2D, fibronectin matrix assembled by 3T3-L1 cells grown in tumor-associated medium was reduced to control levels in the presence of FUD. As shown in Fig. 2C, IL-8 induction by tumor-associated ECM was completely prevented when A549 cells were seeded onto these fibronectin-depleted, decellularized tumor-associated matrices. These data indicate that fibronectin present in the tumor-associated ECM is required for the TLR4-dependent increase in IL-8 release by NSCLC cells. Further studies on the specific contribution of the FnIII-1c region of matrix fibronectin to the release of IL-8 await the development of reagents which directly target the TLR4 agonist activity of FnIII-1c. It should be noted that the absence of fibronectin from the matrix may disrupt other matrix-associated molecules which may be contributing to the production of IL-8. A recent study has shown that serum levels of IL-8 correlate with lung cancer risk 22 and can be diagnostic for lung cancer in patients with chronic obstructive pulmonary disease 23, suggesting that IL-8 is a marker of early disease that could be targeted to prevent the development of advanced disease.

### Activated NF-κB is present in human NSCLC tumors

**Figure 3:**
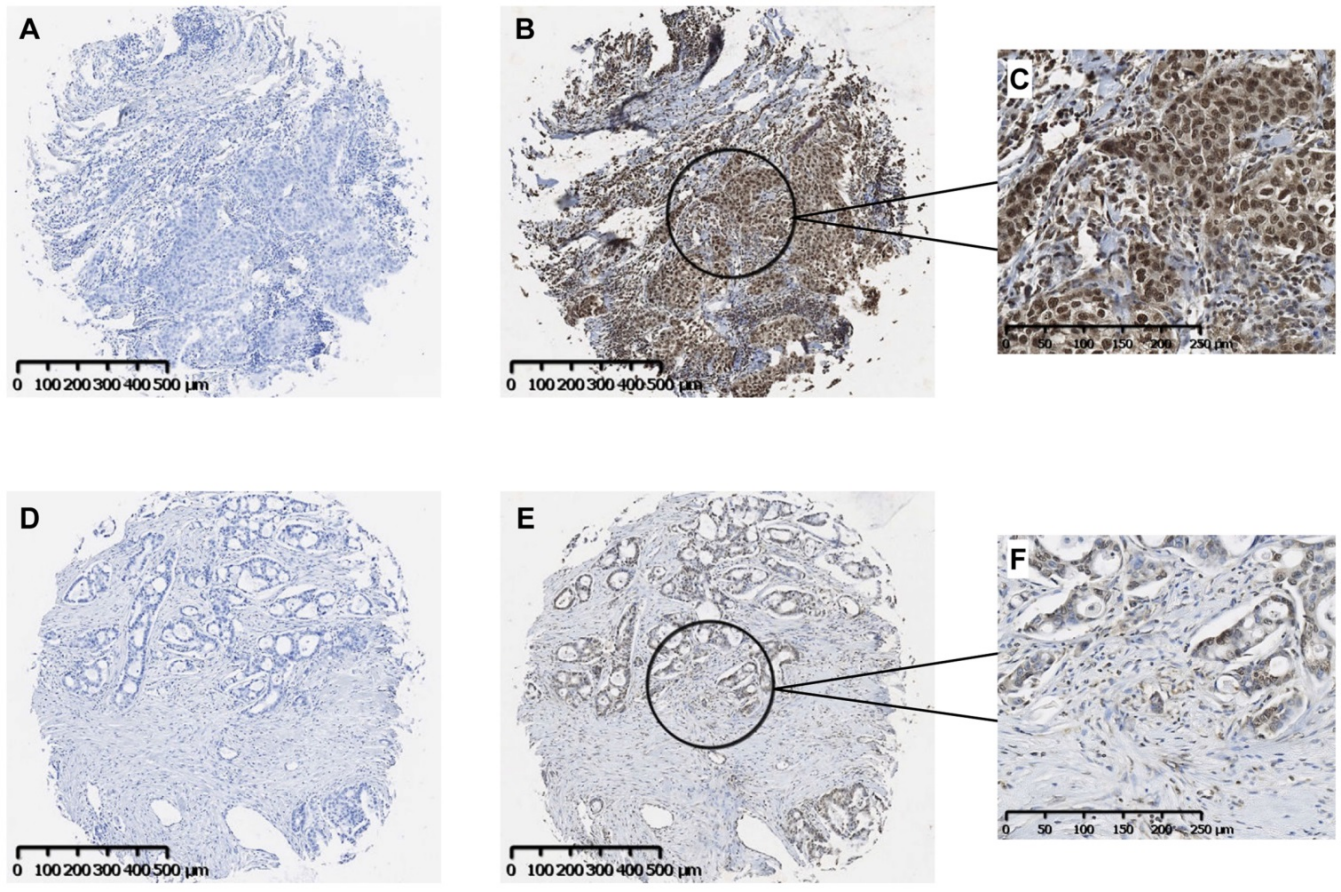
** Immunohistochemical localization of activated NF-κB in human NSCLC tumors.** Representative images of serial sections of squamous cell (**A, B**) or adeno (**D, E**) carcinomas were stained with antibody to the phosphorylated p65 subunit of NF-κB (**B, E**). Controls received no primary antibody (**A, D**). Scale bar = 500 μM. Higher magnifications of the areas designated in **B** and **E** stained images are shown in **C** and **F**. Scale bar = 250 μM.

Our findings suggest that the fibronectin present in the lung tumor stroma contributes to tumor progression by driving a TLR4/NF-κB/IL-8 signaling axis in NSCLC cells. To determine whether NF-κB signaling is active in human lung cancer, we evaluated a panel of human lung tumors for the presence of active NF-κB. As shown in Fig. 3, immunostaining of both squamous cell (Panels B, C) and adenocarcinoma of the lung (Panels E, F) was positive for the phosphorylated NF-κB subunit, p65/RelA. Staining in both tumor types was mostly nuclear with minimal cytoplasmic staining. Control tissues stained with only secondary antibody are shown in Panels A and D. Of the 33 tumors evaluated, 32 (97%) stained positively for p-p65/RelA and 29 (88%) showed nuclear localization. The phosphorylation and nuclear localization of the p65/RelA NF-κB subunit indicate that the NF-κB pathway is active in NSCLC cells from both human squamous cell and adenocarcinomas.

IL-8 mediates resistance of NSCLC to the EGFR kinase inhibitor, Erlotinib. Erlotinib resistant cells convert to a mesenchymal phenotype which is maintained by IL-8 and associated with activation of an NF-κB/p38 pathway. Inhibition of the p38- pathway or suppression of IL-8 synthesis resensitized cells to both Erlotinib and chemotherapies 24,25. Therefore, new therapeutic approaches to block IL-8 signaling may be useful for the treatment of Erlotinib-resistant lung cancers. Our studies suggest that the identification of a fibronectin matrix based signaling pathway, activated through mechanically- induced increases in fibronectin strain may provide novel targets for treatment of drug resistant NSCLC.

## Abbreviations

NSCLC: non-small cell lung cancer
ECM: extracellular matrix
DAMP: damage-associated molecular pattern molecule
TAM: tumor associated matrix
TLR: toll-like receptor
IL-8: interleukin 8
FUD: functional upstream domain of adhesin F1
